# Comparison of Diagnostic Parameters of Acute Coronary Syndromes in Patients with and without Cancer: A Multifactorial Analysis

**DOI:** 10.3390/curroncol31080357

**Published:** 2024-08-20

**Authors:** Anna Ciołek, Grzegorz Piotrowski

**Affiliations:** 1 Department of Cardiooncology, Medical University of Lodz, 90-647 Lodz, Poland; 2 Department of Cardiology, Nicolaus Copernicus Memorial Hospital, Pabianicka 62, 93-513 Lodz, Poland

**Keywords:** cancer, acute coronary syndrome, coronarography

## Abstract

Background: The simultaneous occurrence of cancer and acute coronary syndromes (ACSs) presents a complex clinical challenge. This study clarifies variances in diagnostic parameters among ACS patients with and without concurrent cancer. Methods: This retrospective study included 320 individuals diagnosed with ACS, stratified equally into two cohorts—one with cancer and the other cancer-free. We evaluated risk factors, symptom profiles, coronary angiography results, echocardiographic evaluations, and laboratory diagnostics. Statistical analysis was performed using Student’s *t*-test, the Mann–Whitney U test, and the chi-square test. Results: Cancer patients were older (mean age 71.03 vs. 65.13 years, *p* < 0.001) and had a higher prevalence of chronic kidney disease (33.1% vs. 15.0%, *p* < 0.001) but a lower prevalence of hyperlipidemia (59.7% vs. 82.5%, *p* < 0.001). Chest pain was less frequent in cancer patients (72.5% vs. 90%, *p* < 0.001), while hypotension was more common (41.9% vs. 28.8%, *p* = 0.022). NSTEMI was more common in cancer patients (41.9% vs. 30.6%, *p* = 0.048), while STEMI was less common (20.6% vs. 45.3%, *p* < 0.001). RCA and LAD involvement were less frequent in cancer patients (RCA: 18.1% vs. 30.0%, *p* = 0.018; LAD: 18.8% vs. 30.0%, *p* = 0.026). Conclusions: This study demonstrates differences in the clinical presentation of ACS between patients with and without cancer. Cancer patients were less likely to present with chest pain and more likely to experience hypotension. Additionally, they had a higher prevalence of chronic kidney disease and they were less likely to have hyperlipidemia. These findings highlight the need for a careful approach to diagnosing ACS in oncology patients, considering their distinct symptomatology.

## 1. Introduction

Cardiovascular diseases, including acute coronary syndromes, alongside cancer, represent the main causes of mortality in developed countries, collectively accounting for more than two-thirds of global deaths [1]. Owing to advancements in diagnostics and therapeutics, cancer has become a chronic condition, often requiring extended care for patients. Concurrently, there is an increasing prevalence of cardiovascular diseases among oncological patients [2,3]. Notably, several studies indicate a higher mortality rate from cardiovascular diseases than from malignancies in cancer patients [4,5]. The incidence of acute coronary syndromes in this demographic is on the rise, attributed to the extended survival rates in cancer patients [6]. While cancer and acute coronary syndrome are distinct pathologies, some evidence suggests a link between them, involving shared interactive mechanisms, pathophysiology, and etiology.

It is well documented that cancer and its treatments can induce cardiovascular system impairment. This occurs through diverse mechanisms, ranging from the myocardial toxicity of antineoplastic agents to complex disruptions (e.g., temporary impairment in cellular organelles, proteins, and enzymes), resulting in transient myocardial contractility anomalies [7,8]. The applied radiotherapy is not benign to the cardiovascular system either; its mechanisms, such as DNA damage, oxidative stress, and the release of inflammatory and profibrotic cytokines, accelerate coronary artery disease [9]. Furthermore, cancer induces a persistent pro-thrombogenic state, potentially leading to arterial thromboses, including in coronary vessels [10,11].

Despite these insights, data on the concomitance of cancer and acute coronary syndromes remain sparse. This is attributable to the exclusion of cancer patients from major multicenter cardiovascular studies and the disqualification of cardiovascular patients in oncological research. The developing field of cardio-oncology is endeavoring to close this knowledge gap. Yet, current guidelines and research mostly concentrate on the cardiotoxic effects of anticancer drugs [12]. Therefore, there is a need for extensive analyses of the interplay between cancer and cardiovascular diseases. This article attempts to explain the relationship between acute coronary syndromes and cancer, examining demographic characteristics, risk factors, the dominant type of symptoms, types of acute coronary syndrome, diagnostic testing outcomes, and laboratory data in patients with acute coronary syndrome, both with and without concurrent cancer.

## 2. Materials and Methods

In this study, the minimal required sample size, calculated using G*Power 3.1.9.7 tool [13,14], was determined to be 176 participants to achieve a test power of 0.95 for independent samples and 138 participants for linear correlation.

The study was single-centered. The study cohort comprised 160 individuals diagnosed with cancer who were admitted to our department between 2017 and 2021 due to acute coronary syndrome. The control group consisted of 160 randomly chosen patients without cancer who were admitted for acute coronary syndrome in the same period. The study’s criteria for inclusion in the research group were a prior cancer diagnosis, admission for acute coronary syndrome, and agreement to be hospitalized and treated at our facility. Exclusion factors included no previous cancer diagnosis or acute coronary syndrome, refusal of hospitalization, and incomplete data in medical records. For the control group, inclusion criteria were admission for acute coronary syndrome and consent for hospitalization and treatment. Exclusion factors were similar to those of the study group, namely the absence of acute coronary syndrome, refusal of hospitalization, and incomplete data in medical records, but without the requirement of a cancer diagnosis.

It is important to note that this study was conducted during the COVID-19 pandemic. During this period, our facility did not admit patients infected with the SARS-CoV-2 virus. Patients diagnosed with the virus were redirected to specialized treatment centers for COVID-19. Therefore, SARS-CoV-2 infection was an exclusion criterion for both study and control groups in the conducted research.

A retrospective analysis of hospital discharge records was carried out, and the diagnosis of acute coronary syndrome was established by the prevailing ESC guidelines for this condition [15,16].

The following data were analyzed in all patients: age, gender, smoking status, arterial hypertension, history of stroke, hyperlipidemia, diabetes mellitus, chronic kidney disease, type of acute coronary syndrome (ST elevated myocardial infarction—STEMI; non-ST elevated myocardial infarction—NSTEMI; or unstable angina—UA), the dominant type of symptoms (chest pain, dyspnea, hypotension), coronary angiography results (detection of single-, double-, or triple-vessel disease and the artery responsible for the infarction), echocardiography results (left ventricle ejection fraction—LVEF; left ventricle in systole—LVs; left ventricle in diastole—LVd; left atrium dimension—LAD; left atrium index—LAVI; mitral inflow peak early filling velocity to peak atrial filling velocity ratio—E/A; and mitral inflow peak early filling velocity to mitral annular septal peak early diastolic velocity ratio—E/E’), initial troponin results (positive or negative), and laboratory test results (creatinine; glomerular filtration rate—GFR; alanine transaminase—ALT; and aspartate transaminase—AST).

ACS was defined as “…a spectrum of conditions that include patients presenting with recent changes in clinical symptoms or signs, with or without changes on 12-lead electrocardiogram (ECG) and with or without acute elevations in cardiac troponin (cTn) concentrations. Patients presenting with suspected ACS may eventually receive a diagnosis of acute myocardial infarction (AMI) or unstable angina (UA)…” [17] (p. 11).

STEMI was defined as myocardial infarction with persistent ST-segment elevation (or ST-segment elevation equivalents) on ECG accompanied by acute chest pain (or its equivalent) with troponin elevation [15,16,17].

NSTEMI was defined as myocardial infarction without persistent ST-segment elevation, accompanied by acute chest pain (or its equivalent) with troponin elevation [15,16,17].

UA was defined as “…myocardial ischemia at rest or on minimal exertion in the absence of acute cardiomyocyte injury/necrosis.” This is from [17] (p. 11).

Arterial hypertension was defined as a chronic condition in which blood pressure in the arteries is consistently elevated to SBP ≥140 mmHg and/or DBP ≥90 mmHg and/or a prior diagnosis of hypertension, and/or the use of antihypertensive medications [18].

Hyperlipidemia was defined as the presence of abnormally high levels of lipids or lipoproteins in the blood, as outlined in ESC/EAS guidelines [19], and/or a previous diagnosis, and/or the use of lipid-lowering medications.

Diabetes mellitus was defined as a chronic metabolic disorder characterized by persistent hyperglycemia due to either impaired insulin secretion, insulin action, or both, by ESC/EASD guidelines [20] and/or a prior diagnosis, and/or the use of antidiabetic medications.

Chronic kidney disease (CKD) was defined as a progressive loss of kidney function over time, present >3 months, with health implications, according to the KIDGO guidelines [21].

GFR was calculated using the Cockroft-Gault formula, and troponin levels were determined using the electrochemiluminescence method (ECLIA), diagnostic test manufacturer Roche, Combas Pro e801 analyzer (Manufacturer Roche, Basel, Switzerland), cut-off value < 14 ng/L, 99th percentile cut-off value in acute myocardial infarction diagnostics.

This study aimed to compare demographic characteristics (age, gender), risk factors, and presented symptoms among patients with acute coronary syndrome with and without cancer, analyze differences in the type of ACS (STEMI, NSTEMI, UA) in both patient groups, assess the impact of cancer presence on coronary angiography and echocardiography results in ACS patients, and compare laboratory test results (troponin, renal parameters, ALT, AST, blood count) in ACS patients with and without cancer.

The study protocol was approved by the Ethics Committee of the Medical University of Lodz (permission number RNN/240/23/KE).

Descriptive statistics analysis was conducted to compare patients with ACS and ACS with concomitant cancer. Demographic characteristics, risk factors, dominant type of symptoms, infarction type, echocardiography results, and troponin levels were compared. For the analysis of quantitative variables, Student’s *t*-test and the Mann–Whitney U test were employed, while for qualitative variables, a chi-square test of independence was used. An IBM SPSS Statistics 29 software package was utilized for this purpose.

## 3. Results

Patients with concomitant acute coronary syndrome and cancer were older than individuals without accompanying cancer (71.03 vs. 65.13; *p* < 0.001). Women were more frequently found in the group of patients with acute coronary syndrome and concomitant cancer (60.2%) than men (43.7%); *p* = 0.006.

Risk factors such as smoking, chronic coronary artery disease, hypertension, previous stroke, diabetes, and prior myocardial infarction were similarly prevalent in both groups. Patients with cancer were less likely to have hyperlipidemia (59.7% vs. 82.5%; *p* < 0.001) and more likely to have chronic kidney disease (33.1% vs. 15.0%; *p* < 0.001).

In the case of concomitant cancer, NSTEMI occurred more frequently (41.9% vs. 30.6%, *p* < 0.048), while STEMI occurred less frequently (20.6% vs. 45.3%, *p* < 0.001). There was no statistical difference in the occurrence of unstable angina.

There is a statistically significant difference in the frequency of chest pain between ACS patients with cancer and those without cancer (72.5% vs. 90%; *p* < 0.001). Additionally, there is a statistically significant difference in the occurrence of hypotension between ACS patients with cancer and those without cancer (41.9% vs. 28.8%; *p* < 0.022). However, no statistically significant difference was found in the frequency of dyspnea between the two groups.

The RCA and LAD were more frequently responsible for ACS in patients without cancer (30.0% vs. 18.1%; *p* = 0.018 for RCA and 30.0% vs. 18.8%; *p* = 0.026 for LAD). Single-vessel coronary disease (32.7% vs. 18.1%, *p* = 0.003) and double-vessel coronary disease (33.3% vs. 22.1%, *p* = 0.032) were more common in patients without concomitant cancer.

The E/A ratio shows a median value of 0.90 in MI patients and 0.80 in those with cancer. This difference is statistically significant (*p* = 0.016), suggesting a potential impact of cancer on diastolic function.

Patients with a history of ACS with concomitant cancer were less likely to have a positive troponin value. Furthermore, this group exhibited lower levels of AST and ALT than patients without accompanying cancer (25.50 vs. 28.0; *p* = 0.004 and 21.0 vs. 22.0; *p* < 0.001). However, creatinine levels and GFR were similar in the examined patients, regardless of the presence of concomitant ACS and cancer.

Descriptive data along with statistical significance are presented in Table 1

To summarize the previous analyses and create a more comprehensive picture of patients with ACS and concomitant cancer, logistic regression analysis using backward elimination was performed. Through this process, a well-fitted model was successfully created in the 28th step (Table 2). The explanatory variables included demographic data, comorbidities, risk factors, ACS type, echocardiography results, and troponin levels, with the outcome variable being the presence of concomitant myocardial infarction and cancer. The analyzed model was well fitted to the data (χ^2^(16) = 64.82; *p* < 0.001), explaining a total of 53% of the variance in the outcome variable (R^2^ Nagelkerke = 0.53).

In patients with ACS and cancer, hypertension was relatively present 31% less frequently (odds ratio [OR] = 0.29, 95% confidence interval [CI]: 0.10–0.83); hyperlipidemia was 39% less frequent (OR = 0.61, 95% CI: 0.40–0.93). Conversely, chronic kidney disease was 14 times more prevalent in this group (OR = 14.16, 95% CI: 2.05–97.77), two-vessel coronary artery disease was 5 times more frequent (OR = 4.85, 95% CI: 2.97–7.92), and three-vessel coronary artery disease was 5.5 times more frequent (OR = 5.50, 95% CI: 3.18–9.52). Additionally, patients with cancer exhibited significantly lower troponin levels (OR = 0.05, 95% CI: 0.037–0.067), and the right coronary artery was significantly more often responsible for heart attacks (OR = 0.25, 95% CI: 0.169–0.370).

## 4. Discussion

ACS and cancer are prevalent causes of global mortality. The cardiotoxic effects of oncologic therapies are extensively documented, with vasotoxicity constituting a significant adverse effect. This vasotoxicity starts from chemotherapy-induced damage to the vascular endothelium. Antineoplastic agents can cause both irreversible harm through structural alterations in vascular tissues and temporary disruptions, including reversible vasoconstriction and thrombogenesis [22]. Additionally, cancer and oncology treatments, such as chemotherapy and radiotherapy, can impact the cardiovascular system through endothelial damage, increased vascular reactivity, and thrombosis formation. Radiotherapy can cause myocardial tissue damage through oxidative stress and inflammation, leading to atherosclerosis and myocardial infarctions [7,8,9]. While each pathomechanism may precipitate myocardial ischemia, their clinical manifestations in oncology patients vary from those observed in non-oncologic populations. It is hypothesized that these differences in the etiology of ACS may lead to differences in diagnostic approaches, morphological types of ACS, and patient demographic profiles. The objective of this study was to explain these differences.

It has been indicated that patients with both cancer and acute coronary syndrome tend to be older than those without cancer. Numerous studies have highlighted that as age progresses, the risk of cancer increases, mainly due to DNA mutations accumulating over time. This cancer risk peaks around the age of 70 and then starts to slightly recede. Notably, in 2009, more than half of cancer diagnoses were made for individuals aged 65 and above [23]. In a separate investigation, it was demonstrated that in the United States, over 90% of cancer cases were identified in individuals aged 50 years and above [24]. The median age for the onset of acute coronary syndrome in a presented study in cancer patients was 71. In the subsequent section, we compared the two groups based on risk factors and gender. In our study, we observed a higher number of females with ACS in the cancer group. This may be related to the epidemiology of certain cancers, such as breast cancer, which is one of the most prevalent cancers among women [25]. Furthermore, treatments such as radiotherapy, especially left-sided, due to its anatomical proximity, may increase the risk of developing ACS [26]. An increased incidence of ACS in females with cancer has also been observed in other studies [27]. However, our study did not thoroughly analyze the specific types of cancers affecting the oncological patients, which could have provided more detailed insights into this phenomenon. Further research is necessary to clarify the exact reasons for this gender disparity in ACS incidence among cancer patients. Referring further to Table 1, an analysis of the risk factors indicated that hyperlipidemia and CKD were statistically significant. The relationship between CKD and cancer manifests through diverse pathways. Oncological pathologies can accelerate CKD either directly or indirectly via the adverse effects of therapeutic interventions. Conversely, CKD may act as a predisposing factor for the onset of cancer. The concomitance of these diseases could be attributed to shared etiological factors, including exposure to various toxins [28]. Additionally, the diminished renal function observed in oncological patients may be a consequence of advanced age in this group. Furthermore, the malignancy itself, along with the applied oncologic treatments, might exacerbate renal impairment. The logistic regression analysis demonstrates a substantial increase in the risk of concurrent ACS and cancer in comparison to patients without this condition, particularly in cases involving coexisting CKD, where a 14-fold increase in risk was noted. This underscores the necessity for increased attention in the monitoring and management of oncological patients with such comorbid conditions. The analysis indicates that the incidence of simultaneous myocardial infarction and cancer was observed to be 31% less frequent in hypertensive patients relative to their non-hypertensive counterparts. In a similar vein, individuals diagnosed with hyperlipidemia exhibited a reduction in risk by 39% for the co-occurrence of these conditions. This evidence potentially presumes that conventional therapeutic strategies for these disorders may confer a protective effect against the development of ACS in oncological patients [29,30].

Another important aspect of this study was the comparison of both patient groups in terms of presented clinical symptoms. This study analyzed the main complaints reported during admission to the department by patients experiencing an ACS episode. In our study, the clinical presentation between these two groups differed significantly. Patients with cancer were less likely to present with chest pain compared to those without cancer (72.5% vs. 90%; *p* < 0.001). This difference may be attributed to changes in pain perception among cancer patients. Oncological patients often experience a range of symptoms due to their primary disease and its treatments, which can mask or alter the presentation of typical ACS symptoms. For example, neuropathy induced by chemotherapy and radiotherapy may affect pain perception, leading to a less pronounced presentation of chest pain. Additionally, cancer patients often use high doses of opioid medications to manage pain from their primary disease, which can also modify the typical sensation of myocardial infarction pain [6,31,32,33]. Dyspnea was reported more frequently in cancer patients (57.5% vs. 47.5%; *p* = 0.100), although this difference in our study was not statistically significant. Dyspnea in cancer patients can be attributed to several factors, including pulmonary involvement by metastases or chemotherapy-induced lung injury [32,34]. These additional factors may exacerbate the sensation of breathlessness during an ACS event, making dyspnea a more prominent symptom in this population. Hypotension was significantly more common in patients with cancer (41.9% vs. 28.8%; *p* = 0.022). This increased frequency of hypotension in cancer patients with ACS is also observed in the literature. It could be related to various factors, such as the side effects of cancer treatments (e.g., chemotherapy-induced cardiomyopathy). Cancer-related cachexia and malnutrition may also contribute to the hemodynamic instability observed in these patients [31,33,35]. The different clinical presentation of ACS in patients with cancer suggests the need for increased vigilance when assessing the overall condition of these patients suspected of having ACS. Clinicians should be aware that typical coronary symptoms in this patient group may not be as pronounced, and that cancer patients may not report any pain at the time of examination. This requires a broader consideration of other signs and symptoms reported by cancer patients. In conclusion, the clinical presentation of ACS in patients with cancer differs markedly from those without cancer. The lower prevalence of chest pain, along with higher incidences of dyspnea and hypotension, should prompt clinicians to maintain alert for ACS in cancer patients, even in the absence of typical symptoms.

This study investigated the incidence of hyperlipidemia and the variants of ACS in cohorts with and without a cancer diagnosis. The findings clarify that cancer-afflicted patients concurrently diagnosed with ACS exhibited lower lipid profile indices compared to their cancer-free counterparts. Furthermore, the prevalence of STEMI was notably higher in patients without cancer, whereas those with cancer demonstrated a greater propensity towards NSTEMI. This divergence is likely caused by the various pathogenesis of ACS in the two patient groups. In individuals not suffering from cancer, ACS predominantly originates from atherosclerotic processes, characterized by the accumulation of plaques within coronary arteries. Such plaques are susceptible to rupture, potentially leading to thrombogenesis and the consequent obstruction of myocardial blood flow [36]. Conversely, in the oncological population, ACS can be caused by a multitude of factors including chemotherapy, radiation therapy, and the malignancy itself, all of which may induce damage to coronary arteries, thereby elevating the risk of arterial rupture or stenosis. These distinct pathophysiological mechanisms underlying ACS in cancer and cancer-free patients seemingly contribute to the heightened occurrence of NSTEMI in the former group.

Subsequently, a detailed study was conducted on the outcomes of coronary angiography in the patient cohort. This examination revealed that in individuals without cancer, ACS mostly originated from alterations in the principal coronary arteries, namely the right coronary artery and the left anterior descending artery. The angiographic evaluations frequently indicated the presence of single- and double-vessel disease in these patients. Nonetheless, for an interpretation of these findings, it is recommended that an expanded study encompassing a more substantial participant population be undertaken.

The echocardiographic outcomes in oncology patients indicate a frequent impairment of left ventricular diastolic function. This observation aligns with preceding meta-analyses focusing on breast cancer patients devoid of pre-existing cardiac conditions, which identified deteriorated diastolic function parameters [37]. Notably, the E/A ratio was noted as a potential early marker of these alterations. In this present study, the E/A ratio exhibited a significant divergence in the cancer cohort, although the E/e’ ratio remained unaltered.

The prognostic utility of troponin levels in diagnosing ACS is crucial. However, in the context of patients with cancer and suspected ACS, this utility is not as clear. Due to changes in laboratory procedures that can lead to differences in absolute troponin levels, we only considered whether troponin levels were above or below the normal range. Additionally, various protocols for serial troponin measurements were employed during the diagnosis of ACS (based on corresponding ESC guidelines). Consequently, only the initial troponin measurement was considered, and the final diagnosis of ACS type was based on serial measurements and ECG changes.

It is also noteworthy that in our study, troponin values were not correlated with the type of cancer, its stage, or the treatment administered. Within this study’s framework, the incidence of troponin levels exceeding the normal threshold was less frequent in patients concurrently diagnosed with cancer and coronary artery disease compared to in those without cancer. There may be several reasons that explain this phenomenon. The likelihood of experiencing an STEMI may be reduced in cancer patients. In such a scenario, the extent of myocardial necrosis might be limited, thereby resulting in lower troponin concentrations. Our study also demonstrated that patients with cancer were more frequently diagnosed with UA, although these results were not statistically significant.

On the other hand, elevated high-sensitivity troponin is recognized as a prognostic indicator for mortality from any cause in cancer patients [38]. Additionally, elevated levels have been documented in cancer patients absent of ACS, potentially as a consequence of treatment modalities such as chemotherapy and radiation, which can cause cardiac injury [39]. It has been shown that troponin level assessment can serve as an early detection tool for cardiotoxicity in patients with advanced non-small-cell lung cancer treated with nivolumab [40].

Due to these discrepancies and the limitations of our study (lack of correlation with cancer type and stage), it seems prudent to approach the results concerning troponin levels with caution. There is no doubt that the diagnostic value of measuring cardiac troponin in cancer patients is significant; however, it should be noted that the values may differ somewhat compared to patients without cancer. Comprehensive future research is required to establish the impact of cancer on the dynamics of troponin increase during ACS.

The final variables assessed in this study were renal and hepatic parameters. Concerning hepatic parameters, cancer patients exhibited lower values compared to their cancer-free counterparts. Reduced levels of liver enzymes in cancer patients may be associated with the presence of the malignancy itself. The literature reports that oncological patients may exhibit altered levels (either increased or decreased) of AST and ALT compared to non-cancer patients, potentially due to differences in liver cell metabolism in cancer patients [41]. Furthermore, lower levels of ALT and AST in cancer patients may be attributed to sarcopenia and frailty, which are common conditions in this population and are associated with higher mortality rates [42]. The level of aminotransferases, enzymes predominantly located in the liver, may serve as an independent risk factor for the development of coronary artery disease and ACS [43]. In one study, it was demonstrated that the maximum concentration of liver enzymes strongly correlated with the infarct size, ejection fraction, and microcirculation disorders [44]. It is observed that aminotransferase levels are frequently elevated during acute coronary syndrome episodes, with a correlation to the extent of myocardial necrosis, often resulting from liver damage caused by hypoxia. In the context of extensive myocardial infarction, a diminished cardiac output can adversely affect liver perfusion [45]. This phenomenon might indirectly indicate that in cancer patients experiencing myocardial infarction, the necrotic areas during ischemic episodes are comparatively smaller. However, additional research is warranted to substantiate this observation.

## 5. Limitations

When analyzing the results of our study, it is important to consider several limitations that may affect the interpretation of our findings. First, our sample size was relatively small, which may limit the statistical power and generalization of our results for a wider population. Therefore, there is a risk that some subtle differences may not have been detected.

Another limitation is the lack of long-term follow-up of participants, which means that we were unable to assess long-term effects or variability of outcomes over time. In addition, the research methods used may not have taken into account all the relevant variables, which may affect the reliability and validity of our results.

Individual types of cancer, their respective stages, and the treatments administered were not analyzed about the assessed variables. The authors believe that the type of cancer, its progression, and the applied therapeutic interventions exert a significant impact on the cardiovascular system. The exclusion of these data represents a limitation of the conducted study. Additionally, this is a retrospective analysis, so some data are impossible to obtain.

Despite these limitations, we believe that our study provides important insights into the differences in diagnostic parameters in patients with acute coronary syndrome and cancer and without oncological disease.

## 6. Conclusions

The coexistence of cancer and ACS presents diagnostic challenges for clinicians. Our study revealed several differences in the clinical presentation of ACS between patients with and without cancer. Cancer patients with ACS are less likely to present with chest pain and more likely to exhibit hypotension compared to patients without cancer. Additionally, cancer patients had a higher prevalence of chronic kidney disease and a lower prevalence of hyperlipidemia and elevated liver enzymes. They also had different ECG patterns, with a higher incidence of NSTEMI and lower levels of troponins. These differences highlight the necessity for increased vigilance while diagnosing ACS in oncology patients, taking into account their distinct clinical. Further research is needed in this area to better understand these interactions and relationships.

## Figures and Tables

**Table 1:** The results of the analysis compare patients with acute coronary syndrome with and without concomitant cancer in terms of demographic characteristics, risk factors, the dominant type of symptoms, infarction type, coronarography results, echocardiography results, troponin value, and renal and liver parameters.

	Acute Coronary Syndrome (*n* = 160)	Accompanying Cancer (*n* = 160)	*p*
Demographic characteristics			
Age (years), mean	65.13	71.03	<0.001
Gender (male) (%)	111 (69.4)	86 (53.8)	0.006
Risk factors			
Nicotine addiction (%)	59 (36.9)	48 (30.0)	0.236
Chronic ischemic heart disease (%)	101 (63.1)	99 (61.9)	0.908
Hypertension (%)	125 (78.1)	117 (73.1)	0.362
History of stroke (%)	18 (11.3)	22 (13.8)	0.504
Hyperlipidemia (%)	132 (82.5)	95 (59.7)	<0.001
Diabetes mellitus (%)	45 (28.3)	54 (33.8)	0.333
Chronic kidney disease (%)	24 (15.0)	52 (33.1)	<0.001
Prior myocardial infarction (%)	47 (29.4)	59 (36.9)	0.191
The dominant type of symptoms			
Chest pain (%)	144 (90.0)	116 (72.5)	<0.001
Dyspnea (%)	76 (47.5)	92 (57.5)	0.100
Hypotension (%)	46 (28.8)	67 (41.9)	0.022
Acute coronary syndrome type			
STEMI (%)	72 (45.3)	33 (20.60)	<0.001
NSTEMI (%)	49 (30.6)	67 (41.9)	0.048
UA (%)	38 (23.8)	51 (31.9)	0.134
Coronarography results			
RCA (%)	48 (30.0)	29 (18.1)	0.018
LAD (%)	48 (30.0)	30 (18.8)	0.026
Cx (%)	19 (11.9)	12 (7.5)	0.257
Single-vessel coronary artery disease (%)	52 (32.7)	29 (18.1)	0.003
Two-vessel coronary artery disease (%)	53 (33.3)	34 (22.1)	0.032
Three-vessel coronary artery disease (%)	52 (32.5)	64 (40.0)	0.201
Echocardiography results			
LVEF (%), median (IQR)	51.00 (19.00)	53.00 (17.50)	0.201
LVs (mm), median (IQR)	36.00 (10.00)	35.00 (10.50)	0.239
LVd (mm), median (IQR)	52.00 (11.00)	51.00 (11.00)	0.145
LAD (mm), median (IQR)	43.00 (8.00)	41.00 (11.00)	0.058
LAVi (mL/m^2^), median (IQR)	39.00 (18.50)	37.00 (28.50)	0.398
E/A, median (IQR)	0.90 (0.50)	0.80 (0.50)	0.016
E/E’, median (IQR)	10.85 (5.15)	11.30 (6.30)	0.905
Troponin			
Positive troponin value (%) Renal parameters	147 (91.9)	102 (63.8)	<0.001
Creatinine (mg/dL), median (IQR)	0.93 (0.31)	1.03 (0.54)	0.294
GFR (mL/min/m^2^), median (IQR) Liver parameters	60.00 (0.00)	60.00 (13.00)	0.354
AST (U/L), median (IQR)	28.00 (41.00)	25.50 (34.50)	0.004
ALT (U/L), median (IQR)	22.00 (17.00)	21.00 (19.75)	<0.001

**Table 2:** Logistic regression model explaining the presence of concomitant ACS and cancer based on demographic parameters, comorbidities, risk factors, infarction type, echocardiography results, troponin value, and renal and liver parameters.

	*B*	*SE*	*Wald*(1)	*p*	*OR*
χ^2^(16) = 64.82; *p* < 0.001; R^2^ Nagelkerke = 0.53					
Gender—male	−1.11	0.58	3.68	0.055	0.33
Hypertension	−1.22	0.60	4.16	0.041	0.29
Hyperlipidemia	−1.54	0.69	5.04	0.025	0.21
Chronic kidney disease	2.65	1.14	5.36	0.021	14.16
Two-vessel coronary artery disease	1.58	0.70	5.13	0.024	4.85
Three-vessel coronary artery disease	1.71	0.68	6.21	0.013	5.50
UA	−1.75	1.02	2.95	0.086	0.17
LVd	−0.06	0.03	4.05	0.044	0.94
E/A	0.89	0.50	3.21	0.073	2.43
A positive level of troponin	−2.94	1.10	7.15	0.007	0.05
GFR	0.08	0.04	4.52	0.034	1.08
AST	−0.01	0.01	3.00	0.084	0.99
ALT	0.02	0.01	4.54	0.033	1.02
Infarct-related artery—RCA	−1.40	0.65	4.59	0.032	0.25
Infarct-related artery—Cx	−1.26	0.77	2.69	0.101	0.29
Constant	6.35	3.23	3.86	0.049	570.98

Annotation. Dependent variable: type of myocardial infarction—no concomitant tumor (0); concomitant tumor (1). *B*—regression coefficient; *SE*—standard error; *Wald*(1)—Wald(1) test result; *p—p*-value, statistical significance; *OR*—odds ratio.

## Data Availability

The datasets presented in this article are not readily available because the data are part of an ongoing study. Requests to access the datasets should be directed to the corresponding author.
